# Variable selection for large *p *small *n *regression models with incomplete data: Mapping QTL with epistases

**DOI:** 10.1186/1471-2105-9-251

**Published:** 2008-05-29

**Authors:** Min Zhang, Dabao Zhang, Martin T Wells

**Affiliations:** 1 Department of Statistics, Purdue University, West Lafayette, IN 47907 USA; 2 Department of Biological Statistics and Computational Biology, Cornell University, Ithaca, NY 14853 USA

## Abstract

**Background:**

Identifying quantitative trait loci (QTL) for both additive and epistatic effects raises the statistical issue of selecting variables from a large number of candidates using a small number of observations. Missing trait and/or marker values prevent one from directly applying the classical model selection criteria such as Akaike's information criterion (AIC) and Bayesian information criterion (BIC).

**Results:**

We propose a two-step Bayesian variable selection method which deals with the sparse parameter space and the small sample size issues. The regression coefficient priors are flexible enough to incorporate the characteristic of "large *p *small *n*" data. Specifically, sparseness and possible asymmetry of the significant coefficients are dealt with by developing a Gibbs sampling algorithm to stochastically search through low-dimensional subspaces for significant variables. The superior performance of the approach is demonstrated via simulation study. We also applied it to real QTL mapping datasets.

**Conclusion:**

The two-step procedure coupled with Bayesian classification offers flexibility in modeling "large p small n" data, especially for the sparse and asymmetric parameter space. This approach can be extended to other settings characterized by high dimension and low sample size.

## Background

With the advent of high-throughput biotechnologies to genotype dense molecular markers throughout the genome, statistical methodologies are crucial in understanding the genetic architecture of complex traits, and in locating genes underlying important traits. Since the pioneering statistical work by Lander and Botstein [1], much effort has been devoted to improving the efficiency and accuracy of QTL mapping. Traditional approaches to QTL mapping test each of dense grid loci on chromosomes via the likelihood ratios of linear regression models (see the reviews by Doerge et al. [2] and Broman and Speed [3]), and Wang et al. [4] also proposed a Bayesian shrinkage estimation of QTL parameters allowing varying shrinkage factors across different effects.

Epistases (that is, interactions between genes) are ubiquitous in biological systems [5] and may even play a more important role than additive effects, as have been shown in human population [6,7] and other organisms [8-12]. However, even a moderate number of markers implies a large number of pairwise combinations, thus creating statistical issues in QTL mapping. Due to the small sample sizes and the lack of efficient statistical tools, the number of identified genes is limited although the existence of epistasis has been recognized for nearly a hundred years [13]. To detect epistatic effects, Kao and Zeng [14] proposed modeling epistasis via orthogonal contrast scales using Cockerham's model; Yi and Xu [15] developed a Bayesian method to detect epistasis using reversible jump Markov chain Monte Carlo (MCMC) algorithm; Yi et al. [16-18] then proposed a Bayesian model selection approach to detect genome-wide epistasis (with the software described in [19]); Bogdan et al. [20] modified Bayesian information criterion (mBIC) to permit the identification of additive effects as well as pairwise interactions; and Cui and Wu [21] also proposed a statistical framework to detect genetic interactions derived from different genomes in self-pollinated plants. Recently, *Żak *et al. [22] developed a rank-based model selection and Shi et al. [23] developed a LASSO-type penalized likelihood method to locate interacting QTL while Bogdan et. al [24] extended mBIC for strongly correlated markers and multiple interval mapping.

Consider *Y*_*i *_as the trait value of strain *i *= 1, ⋯, *n*, and let *X*_*ij *_be the genotypic value of marker *j *= 1, ⋯, *p*_*β *_within the *i*-th strain. Here we focus on the populations with binary markers *X*_*ij *_(coded as -0.5 and 0.5), such as doubled-haploid, backcross or recombinant inbred lines. With available markers (either observed or imputed) densely located on chromosomes, we assume the putative QTL co-transmit with some of the markers. Let I{*X*} denote the set including all pairwise epistases of interest, and define *Z*_*ij *_= *X*_*ik *_*X*_*il *_for the *j*-th candidate epistasis (*k*, *l*) ∈ I{*X*}, *j *= 1, ⋯, *p*_*γ*_. We investigate the additive effects of putative QTL and the epistatic interactions between them through the following multiple regression model,

(1) $$\begin{array}{cc} Y_{i}=\mu +\sum_{j=1}^{p_{\beta }}\beta_{j}X_{ij}+\sum_{j=1}^{p_{\gamma }}\gamma_{j}Z_{ij}+\varepsilon_{i}, & \varepsilon_{i}\overset{iid}{\underset{}{\sim }}N(0,\sigma_{\varepsilon }^{2}), \end{array}$$

where *μ *is the overall mean, *β*_*j *_is the additive effect of marker *j*, *γ*_*j *_represents the *j*-th epistatic effect, and *ε*_*i *_is the random error.

QTL mapping with this multiple regression model can be viewed as a model selection procedure [3,25-27]. However, several characteristics of the data complicate the application of classical statistical methodologies. First, a large amount of missing molecular markers, due to failure in genotyping or selective genotyping, is common in practice. When markers are sparse, the missing genotype information between markers must be inferred. Second, the molecular markers in the same linkage group may be highly correlated. Third, the total number of molecular markers and putative epistases, i.e., *p *= *p*_*β *_+ *p*_*γ*_, is usually much larger than the sample size *n*. Because of these issues, the efficiency and accuracy are usually compromised for easy development of statistical approaches. Characteristics of the "large *p *small *n*" data with missing values require further attention via extensions of traditional model selection approaches. We extend the Bayesian classification approach in Zhang et al. [28] to map QTL with epistases. Spike and slab priors have been used by, for example, Mitchell and Beauchamp [29], George and McCulloch [30], and Ishwaran and Rao [31] to develop Bayesian variable selection approaches. The spike and slab priors consist of two components, with one modeling zero coefficients and the other modeling non-zero ones.

Furthermore, the mixing weight plays a crucial role in condensing the searchable parameter space and enforcing a stochastic search within low-dimensional spaces. When only a limited number of covariates are being investigated, a uniform distribution on [0, 1] or even a fixed value (e.g., 0.5) is usually chosen for the mixing weight. However, when *n *≪ *p*, it is unrealistic to expect half of the variables to be selected because the final model may still be unidentifiable. Instead, we expect that, for a successful variable selection, the prior distributions of the mixing weights depend on both *n *and *p*.

We investigate the predictability of a model developed for a dataset of sample size *n*, and tackle the aforementioned issues. We then construct a two-step Bayesian variable selection approach for model (1) in the case that *n *≪ (*p*_*β *_+ *p*_*γ*_). In the first step, we employ a restrictive prior for each of the coefficients in model (1) in order to enforce stochastic filtering of the large number of candidate variables. This prior also allows flexibility for the possible different numbers and/or scales of positive and negative coefficients (see [32] for more details on its advantage over symmetric priors). A Gibbs sampling algorithm is developed to compute the posterior distributions and to implement the stochastic search. Only a limited number of variables are filtered to go through the second step, which repeats the first step but with much fewer candidate variables. The second step is necessary to model (1) when *n *≪ (*p*_*β *_+ *p*_*γ*_), as the priors in the first step could potentially be too restrictive. The performance of our approach is evaluated via a simulation study and application to real datasets.

## Results and Discussion

### Simulation

Simulation studies were performed to evaluate the performance of our method in the case of *p *≫ *n*. We simulated 56 markers across 3 chromosomes, with each having 10, 20, and 26 markers, and being 56.7 cM, 133.5 cM and 171.6 cM long respectively. We specify $\sigma_{\varepsilon }^{2}$ = 0.5415, and the locations of 28 markers are chosen based on the Drosophila data [28], which include 221 inbred introgression lines between two closely related species. The other 28 markers are chosen such that the neighboring markers are at least 5 cM away. Table 1 shows the detailed information of the non-zero effects specified in the simulation, including two additive effects and three epistatic effects. To assess whether our method is able to identify different types of epistatic effects, we include all three possible interactions in the simulation: (1) neither of the two markers has additive effects (that is, 2–133.8 and 3–56.7); (2) one of them has additive effects (that is, 1–24.7 and 2–47.8); (3) both have additive effects (that is, 2–47.8 and 3–141.5). All epistatic effects were set at the same size to avoid its effects on detectability. Due to the intensive computation involved in Gibbs sampling, a total of 100 complete data sets were simulated. Each of the 100 data sets was analyzed using two models, one model with both additive and epistatic effects while the other with additive effects only. When mapping QTL with epistases, we have a total number of 1596 variables (56 additive-effect loci and 1540 epistases) versus 221 observations in the model.

**Table 1:** Design of the simulation studies.

Type of effect	Marker(s)	Effect size	Heritability
Additive effect	2–47.8	0.8	0.0989
	3–141.5	1.2	0.2225

Interaction effect	(1–24.7, 2–47.8)	1.7321	0.1159
	(2–47.8, 3–141.5)	1.7321	0.1159
	(2–133.8, 3–56.7)	-1.7321	0.1159

Each marker is referred by its chromosome index and its location on the chromosome.

For the model without epistases, both markers can be detected in most of the 100 simulated datasets even when the false discovery rate (FDR) is controlled as low as 0 (via setting the Bayes factor higher than 3.2), see Table 2. When modeling the epistases, all (additive and interaction) effects are still detected in more than 90% of the data sets for all levels of Bayes factor (BF) though the FDRs are higher. For those data sets with any effect not identified, the immediate neighbors of the corresponding marker locus are mostly detected instead. As expected, it is more difficult to detect epistases than to detect additive effects. The epistasis of markers both having additive effects is the easiest to be detected among all epistases. The true parameter values are included in their 95% credible intervals with the associated posterior probabilities being very close to one (results not shown).

**Table 2:** Simulation results on the basis of model (1).

Model	Marker(s)	Mean	SE	BF ≥ 1	BF ≥ 3.2	BF ≥ 10	BF ≥ 100
Without Epistases	2–47.8	0.7453	0.1340	94 (5)	93 (5)	92 (5)	90 (3)
	3–141.5	1.1222	0.231	100 (0)	100 (0)	100 (0)	100 (0)
	FDR (additive)	--	--	0.0067	0	0	0

With Epistases	2–47.8	0.7610	0.1439	94 (6)	93 (6)	93 (6)	93 (6)
	3–141.5	1.1316	0.1402	100 (0)	100 (0)	100 (0)	100 (0)
	(1–24.7, 2–47.8)	1.5607	0.3921	92 (6)	91 (7)	91 (7)	90 (7)
	(2–47.8, 3–141.5)	1.5558	0.3054	97 (3)	96 (4)	96 (4)	96 (4)
	(2–133.8, 3–56.7)	-1.6204	0.3875	92 (8)	92 (8)	92 (8)	90 (10)
	FDR (additive)	--	--	0.0408	0.0333	0.0133	0.0067
	FDR (epistatic)	--	--	0.4872	0.3251	0.2283	0.1122

Out of 100 simulated data sets, the total numbers of data sets that correctly identify the true additive and interaction effects (in the brackets, their neighboring ones when the true ones are missed) are counted respectively when thresholding the Bayes factor (BF) at different levels. Also listed are the mean and standard error (SE) of the estimated effect sizes.

## Application

We apply the developed method to the *simulans *backcross II (BS2) data and the *mauritiana *backcross II (BM2) data [33,34]. An F_1 _population was first produced by females from an inbred line of *D. simulans *and males from an inbred line of *D. mauritiana*. Then the F_1 _females were backcrossed to the parental line of *D. simulans*, which was fixed for different alleles at 45 marker loci, to produce a *simulans *backcross (BS) population. A *mauritiana *backcross (BM) population was also produced by backcrossing the F_1 _females to the other parental line. Based on the two different times of crossing, a total of four data sets were obtained, namely, BS1 (*n *= 186), BS2 (*n *= 288), BM1 (*n *= 192), and BM2 (*n *= 299). The phenotypic value of an individual is a morphometric descriptor of the posterior lobe, obtained by averaging both sides of the first principal component (PC1) of the Fourier coefficients of the posterior lobe. The genotypes of males were determined at each marker locus, and genetic map positions were estimated from gametes produced by the F_1 _females in this study. Further information about the data is referred to Liu et al. [33] and Zeng et al. [34].

Employing multiple interval mapping (MIM) [25,35] to the BS2 data, Zeng et al. [34] detected a total of 16 additive effects and no epistatic effect. Pooling all four data sets, Zeng et al. [34] detected three extra additive effects and six epistatic effects. These epistatic effects appeared to be relatively unimportant for PC1 in the interspecific backcross populations, which carried an observation difficult to interpret biologically. Of the 19 additive effects, 18 additive effect estimates have the same sign [34]. Zeng et al. [34] explained this interesting phenomena as an unusually strong directional selection, although Tanksley [36] suggested that transgressive segregation usually followed a mixture of plus and minus alleles in each species as demonstrated by most previous analyses of quantitative traits.

We focused our analysis on the BS2 and BM2 data with the standardized phenotypic values. Of the 19 putative QTL reported by Zeng et al. [34], only nine are at least 1 cM away from the 45 marker loci. Therefore, we analyzed both datasets with these 54 additive effects (nine putative QTL and 45 markers) and all possible pairwise interactions (that is, 1431 putative epistases). When controlling BF ≥ 1, the analysis of the BS2 data reported a total of 25 additive effects (see Table 3), including all nine putative QTL, but no epistatic effect. The analysis of the BM2 data instead reported a total of 20 additive effects (see Table 4), including three of the nine putative QTL, and 18 epistatic effects (see Table 5). On the basis of the simulation study, we may expect less than 0.67% FDR for those 17 and 16 additive effects reported with BF ≥ 100 in analyzing the BS2 and BM2 data respectively. Similarly, three epistatic effects reported in analyzing the BM2 data have BF ≥ 100, less than 12% of which may be false discoveries.

**Table 3:** Additive effects with BF ≥ 1 in analyzing the BS2 data.

Marker	Coefficient	S.D.	BF
1–3.6	-0.3797	0.0707	> 1000
1–23.4	-0.3462	0.0426	> 1000
2-0	-0.2284	0.0493	> 1000
2–17.08	-0.1906	0.1055	> 1000
2–27	-0.1262	0.1491	> 1000
2–28.53	-0.1618	0.1387	> 1000
2–69	-0.2969	0.1382	> 1000
2–113.92	-0.0682	0.0487	4.38
2–143	-0.0454	0.0592	1.72
2–145.85	-0.0322	0.0648	1.29
3-0	-0.1726	0.0880	> 1000
3–21.3	-0.3100	0.0569	> 1000
3–43.2	-0.1482	0.1052	> 1000
3–47	-0.1261	0.1571	> 1000
3–49.99	-0.2164	0.0992	> 1000
3–75	-0.4018	0.1072	> 1000
3–94	-0.2147	0.1360	> 1000
3–101.29	-0.0520	0.0904	2.03
3–117	-0.0941	0.0960	29.55
3–126.62	-0.0378	0.0780	1.20
3–134.6	-0.0724	0.1255	4.21
3–139	-0.2624	0.1604	> 1000
3–147.69	-0.0420	0.0833	1.29
3–160	-0.1847	0.1154	> 1000
3–171.22	-0.3295	0.0567	> 1000

The position of each significant additive effect is specified by an index of the corresponding chromosome and its location on this chromosome (cM). The estimated sizes of additive effects and the standard deviations of the Markov chains are also shown in the columns of coefficient and S.D., respectively.

**Table 4:** Additive effects with BF ≥ 1 in analyzing the BM2 data.

Marker	Coefficient	S.D.	BF
1-0	-0.2181	0.1426	> 1000
1–3.6	-0.1438	0.1506	920.66
1–23.4	-0.1909	0.0654	> 1000
2–6.98	-0.2393	0.0809	> 1000
2–27	-0.3361	0.0855	> 1000
2–67.96	-0.0561	0.1093	1.29
2–69	-0.1473	0.1146	> 1000
2–113.92	-0.2496	0.0509	> 1000
2–145.85	-0.1145	0.0856	79.06
3–4.99	-0.1973	0.0954	> 1000
3–14.33	-0.2855	0.0928	> 1000
3–28.74	-0.1754	0.0934	> 1000
3–43.2	-0.0586	0.1077	1.80
3–47	-0.2213	0.1648	> 1000
3–49.99	-0.1749	0.1355	> 1000
3–83.15	-0.5978	0.0781	> 1000
3–126.62	-0.1970	0.1066	> 1000
3–147.69	-0.0698	0.0826	3.05
3–161.43	-0.1982	0.0950	> 1000
3–171.22	-0.2385	0.1028	> 1000

The position of each significant additive effect is specified by an index of the corresponding chromosome and its location on this chromosome (cM). The estimated sizes of additive effects and the standard deviations of the Markov chains are also shown in the columns of coefficient and S.D., respectively.

**Table 5:** Epistatic effects with BF ≥ 1 in analyzing the BM2 data.

Markers	Coefficient	S.D.	BF
(1–3.6, 3–14.34)	0.0601	0.1077	1.36
(1–3.6, 3–101.29)	0.0383	0.0994	1.05
(1–14.2, 2–28.53)	0.0156	0.0792	1.07
(1–14.2, 3–134.6)	0.2231	0.1552	116.30
(1–14.2, 3–139)	0.1689	0.1453	13.40
(2–17.08, 3–157.73)	0.3304	0.0806	> 1000
(2–28.53, 3–101.29)	0.2688	0.1063	> 1000
(2–34.72, 3–76.3)	0.1307	0.0960	5.34
(2–113.92, 3–83.15)	0.0779	0.0911	1.89
(2–138.82, 3–147.69)	0.1678	0.0943	12.23
(2–143, 3–101.29)	0.0463	0.0972	1.25
(2–145.85, 3–28.74)	0.0896	0.0909	2.61
(2–145.85, 3–43.2)	0.0330	0.0980	1.07
(2–145.85, 3–101.29)	0.0419	0.0921	1.29
(3–21.3, 3–76.3)	0.0797	0.0856	1.97
(3–28.74, 3–53.54)	0.0487	0.0999	1.19
(3–43.2, 3–123.32)	0.0400	0.1014	1.04
(3–53.54, 3–123.33)	0.1925	0.1226	43.36

The QTL positions of each significant epistatic effect are specified by the indices of the corresponding chromosomes and the locations on the chromosomes (cM). The estimated sizes of the epistatic effects and the standard deviations of the Markov chains are also shown in the columns of coefficient and S.D., respectively.

Interestingly, the 25 additive effects detected from the BS2 data include all those detected by Zeng et al. [34] except the 2–135, 3–5 and 3–83 (we consider the markers within 1 cM to be same), but the 20 additive effects detected from the BM2 data only include nine of those detected by Zeng et al. [34]. On the other hand, nine additive effects (i.e., 2–28.53, 2–145.85, 3-0, 3–43.2, 3–49.99, 3–101.29, 3–126.62, 3–134.6, 3–147.69) from the BS2 data are not reported by Zeng et al. [34], and eleven additive effects from the BM2 data (i.e., 1-0, 2–6.98, 2–67.96, 2–145.85, 3–14.33, 3–28.74, 3–43.2, 3–49.99, 3–126.62, 3–147.69, 3–161.43) are not reported by Zeng et al. [34]. Note that almost each additive effect uniquely detected by Zeng et al. [34] has a neighboring one (within 10 cM) in our lists except 2–135 and 3–94 for the BM2 dataset, and almost each additive effect unique in our lists has a neighboring one (within 10 cM) detected by Zeng et al. [34]. Per the discussion on the precision of QTL location by Bogdan and Doerge [37] and Bogdan et al. [24], these effects of close neighbors may be due to identical QTL. Our analysis reported *R*^2 ^= 0.934 and *R*^2 ^= 0.902 for the BS2 and BM2 data respectively.

## Conclusion

This article extends the Bayesian framework in Zhang et al. [28] to identify both additive and epistatic effects of QTL based on model (1). The advantage of this approach mainly lies in the flexible priors for the regression coefficients by accounting for some characteristics of "large *p *small *n*" data, the predictability of a model constructed with size *n *data, and the two step strategy for dimension reduction. A Gibbs sampler is developed to draw Markov chain samples from the posterior distributions, which can be considered as a stochastic search for an optimal model. Unlike information criteria based model selections which require calculation of the effective sample size for incomplete data, missing values can be naturally imputed within the Gibbs sampling scheme. The corresponding algorithm has been implemented in Matlab and is available as QTLBayes .

Bayesian variable selections can be viewed as penalized likelihood approaches, which have been studied recently [38,39]. With "large *p *small *n*" data, it is not clear how to set up the penalty properly such that it will neither overpenalize nor underpenalize the likelihood. An overpenalized likelihood will lose some significant variables of particular interest, while an underpenalized likelihood will introduce false positives. The predictability of size *n *data sheds light on the choice of this penalty. Since a size *n *data set will allow us to understand the variation of the trait explained by only *p*_*n *_= *O*($\sqrt{n}$) QTL with accuracy *O*(*n*^-1/2^), selecting too many variables into the model will ruin this practice of QTL mapping. As shown by Bogdan and Doerge [37], severely biased estimates can be resulted from large genome and/or marker number in QTL mapping. We propose a Bayesian framework to resolve the bias problem. We have illustrated our approach by application to the BS2 and BM2 data [33,34], both of which have 45 markers observed across three chromosomes. The disadvantage of this approach is the heavy computation involved as the computation-intensive Markov chain Monte Carlo algorithm is utilized. For example, the analysis of a dataset with more than 200 markers from 1,000 subjects take almost 24 hours using one Intel^® ^Xeon™ CPU at 2.80 GHz.

Coding binary markers with -0.5 and 0.5 has been commonly utilized in QTL mapping as it does not introduce correlation between additive effects and interactive effects, and such uncorrelation benefits the identification of additive effects. On the other hand, coding binary markers with 0 and 1 introduces correlation and thus is not preferred for QTL mapping with epistases [40,41]. Although developed for QTL mapping, this approach is completely general and can be applied to other settings with "large *p *small *n*" data, such as associating genomic features to clinical outcomes or phenotypes of biological interest. Unlike QTL mapping data with known missing structure from the linkage information, genomic data with imaging and microarray may require more information to impute missing values because of the unknown missing mechanism. Even though the missing values are usually imputed with a nearest-neighbor approach [42], Gibbs samplers allow natural multiple imputation under the assumption of missing at random (MAR, see Little and Rubin, [43]).

## Methods

### Predictability and Sample Size

Suppose, for a sample of size *n*, we select up to *p*_*n *_(assuming *p*_*n *_<*n*) significant variables into the following regression model,

$$\begin{array}{cc} {\text{Y}}_{n}={\text{X}}_{n}\beta +\varepsilon_{n}, & \varepsilon_{n}\sim N(0,\sigma_{\varepsilon }^{2}I_{n}), \end{array}$$

where ***Y***_*n *_is an *n*-dimensional column vector; ***X***_*n *_is an *n × p*_*n *_design matrix such that $X_{n}^{T}X_{n}=n\times I_{p_{n}}$. The best linear unbiased estimator (BLUE) of *β *is

$${\hat{\beta }}_{n}=\beta +\frac{1}{n}X_{n}^{T}\varepsilon_{n}.$$

Let $\tilde{x}=({\tilde{x}}_{1},{\tilde{x}}_{2},\cdots ,{\tilde{x}}_{p_{n}})$ include *p*_*n *_predictors for $\tilde{y}$ such that $\max_{1\leq j\leq p_{n}}|{\tilde{x}}_{j}|=O(1)$. Since trace$\{Var({\hat{\beta }}_{n})\}=\frac{p_{n}}{n}\sigma_{\varepsilon }^{2},\tilde{x}\beta$ can be consistently estimated by $\tilde{x}{\hat{\beta }}_{n}$. When using $\tilde{x}{\hat{\beta }}_{n}$ to predict $\tilde{y}$, the mean squared prediction error is

$$E[{\left(\tilde{y}-\tilde{x}{\hat{\beta }}_{n}\right)}^{2}]=\sigma_{\varepsilon }^{2}+\frac{p_{n}}{n}\frac{\tilde{x}{\tilde{x}}^{T}}{p_{n}}\sigma_{\varepsilon }^{2}.$$

If *p*_*n *_= *o*(*n*), the mean squared prediction error asymptotically achieves the minimum variance, and thus the prediction is asymptotically efficient.

This illustration implies that, with a sample of size *n *and *p*_*n *_= *O*($\sqrt{n}$) predictors, the mean squared prediction error can reach the minimum prediction error at rate *O*(*n*^-1/2^). Suppose that all *p*_*n *_significant variables could be perfectly selected out of *p *candidates, we still need *p*_*n *_= *o*(*n*) in order to have a chance to correctly understand the variation of the dependent variable explained by the selected predictors. Therefore, we always assume that there are at most *p*_*n *_= *O*($\sqrt{n}$) significant variables among a total of *p *candidates in the case of *p *≫ *n*. Indeed, the study of consistency in a triangular array setting for regression problems was conducted by Huber [44-46]. In examining the underlying theory of 'model-selection' and 'variable-selection' procedures that choose *p*_*n *_explanatory variables from an initial set of variables, Greenshtein and Ritov [46] proved that one may expect consistency for the choice of *p*_*n *_with an order between *o*($\sqrt{n/\log(n)}$) and *o*(*n/*log(*n*)). Our choice of *p*_*n *_= *O*($\sqrt{n}$) satisfies the Greenshtein and Ritov [46] conditions for consistency.

### Bayesian Variable Selection

Here we propose a two-step Bayesian variable selection approach to map QTL with epistases through model (1). With the following Bayesian framework, we first select *c*$\sqrt{n}$ out of *p*_*β *_additive effects and *c*$\sqrt{n}$ out of *p*_*γ *_epistatic effects (e.g., we use *c *= 2), respectively, using a restrictive prior for each coefficient. We then apply the same Bayesian framework to stochastically select the filtered variables, using a non-restrictive prior for each coefficient. Gibbs sampling algorithms are developed to stochastically search low-dimensional subspaces, as implied by the predictability of a size *n *data set.

#### Prior Specification

For a two-state marker system, both additive effects *β*_*j*_, *j *= 1, ⋯, *p*_*β*_, and epistatic effects *γ*_*j*_, *j *= 1, ⋯, *p*_*γ*_, are the primary focus of QTL mapping. As is often the case *p *= (*p*_*β *_+*p*_*γ*_) ≫ *n*, many of these coefficients are zero, either because the variation of the trait can be explained by only a few QTL or because the limited sample size precludes selecting too many variables (otherwise the constructed model is not reliable as shown in the previous section). It is also possible that the number and/or scale of the positive coefficients may be different from those of the negative ones. To account for these properties, a three-component mixture prior is specified for each coefficient *β*_*j *_or *γ*_*j*_. More specifically,

(2) $$\{\begin{array}{l} \beta_{j}\overset{iid}{\underset{}{\sim }}(1-w_{\beta +}-w_{\beta -})\delta (\cdot )+w_{\beta +}N_{+}(\mu_{\beta +},\sigma_{\beta +}^{2})+w_{\beta -}N_{-}(\mu_{\beta -},\sigma_{\beta -}^{2}),\\ \gamma_{j}\overset{iid}{\underset{}{\sim }}(1-w_{\gamma +}-w_{\gamma -})\delta (\cdot )+w_{\gamma +}N_{+}(\mu_{\gamma +},\sigma_{\gamma +}^{2})+w_{\gamma -}N_{-}(\mu_{\gamma -},\sigma_{\gamma -}^{2}), \end{array}$$

where *δ *(·) is a Dirac function with mass one at zero; *N*_+_(*μ*, *σ*^2^) and *N*_-_(*μ*, *σ*^2^) positively and negatively truncate the normal distribution, i.e., *N*(*μ*, *σ*^2^), respectively. Therefore, *w*_*β*+ _(or *w*_*β*-_) is the probability for any single marker, and *w*_*γ*+ _(or *w*_*γ*-_) is the probability for any pair of markers in I{*X*}, to have positive (or negative) interactive effect on the trait.

The hyperparameters, $\sigma_{\beta +}^{2},\sigma_{\beta -}^{2},\sigma_{\gamma +}^{2}$ and $\sigma_{\gamma -}^{2}$, are assumed to have priors as inverse gamma distributions, that is, *IG*(*θ*_*β*+_, *φ*_*β*+_), *IG*(*θ*_*β*-_, *φ*_*β*-_), *IG*(*θ*_*γ*+_, *φ*_*γ*+_), and *IG*(*θ*_*γ*-_, *φ*_*γ*-_), respectively (e.g., setting *θ*_*β*+ _= *θ*_*β*- _= *θ*_*γ*+ _= *θ*_*γ*- _= 0.1 and *φ*_*β*+ _= *φ*_*β*- _= *φ*_*γ*+ _= *φ*_*γ*- _= 10). As a result, the prior on *β *(and *γ*) is essentially a mixture of a point mass at zero and some truncated *t*-distributions, which shrinks the smaller effects towards zero and allows sufficient flexibility for non-zero effects. Furthermore, *t*-type prior distributions yield Bayes rules with desirable decision-theoretic frequentist properties [47]. The hyperparameters, *μ*_*β*+_, *μ*_*γ*+_, *μ*_*β*- _and *μ*_*γ*-_, are assumed to have diffuse priors, and the prior distribution for $\sigma_{\varepsilon }^{2}$ is proportional to 1/$\sigma_{\varepsilon }^{2}$.

As suggested by the predictability of a size *n *data set, we expect to select at most *p*_*n *_= *O*($\sqrt{n}$) out of the *p *variables for the final model. Therefore, we specify the priors for (*w*_*β*+_, *w*_*β*-_) and (*w*_*γ*+_,*w*_*γ*-_) as

(3) $$\begin{array}{cc} w_{\beta +}+w_{\beta -}\sim U(0,c\sqrt{n}/p_{\beta }), & w_{\gamma +}+w_{\gamma -}\sim U(0,c\sqrt{n}/p_{\gamma }) \end{array},$$

that is, expecting at most *c*$\sqrt{n}$ significant additive effects and epistatic effects, respectively. Gaffney [48] and Yi et al. [17], among others, employed similar ideas to rescale the priors based on the number of possible effects. Apparently, when *n *≪ (*p*_*β *_+ *p*_*γ*_), either *c*$\sqrt{n}$/*p*_*β *_or *c*$\sqrt{n}$/*p*_*γ *_is very small, which implies a restrictive prior on each corresponding coefficient. Therefore, we usually select *c*$\sqrt{n}$ additive effects and *c*$\sqrt{n}$ epistatic effects during the first run of Bayesian analysis. We then apply the same Bayesian analysis to these pre-selected variables. The second run of Bayesian analysis has both *w*_*β*+ _+ *w*_*β*- _and *w*_*γ*+ _+ *w*_*γ*-_, *a priori*, uniformly distributed on [0, 1].

#### Likelihood

Let ***Y***_*n *_be the column vector including the trait values of all strains under investigation, let ***X***_*i *_be the vector of all marker values of the *i*-th strain and $X_{n}={\left(X_{1}^{T},\cdots ,X_{n}^{T}\right)}^{T}$, and let ***Z***_*i *_be the vector of all epistatic candidate values of the *i*-th strain. Denote the marginal distribution of *A *as [*A*], and the conditional distribution of *A *given *B *as [*A|B*]. With data (**Y**_*n*_, **X**_*n*_) and the prior specification in Section 3.1, we have the likelihood function, that is, the joint distribution function of the data (***Y***_*n*_, ***X***_*n*_), the parameters (*μ*, ***β***, ***γ***), $\sigma_{\varepsilon }^{2}$, and all hyperparameters

(4) $$\begin{array}{l} (w_{\beta +},w_{\beta -},w_{\gamma +},w_{\gamma -},\mu_{\beta +},\mu_{\gamma +},\sigma_{\beta +}^{2},\sigma_{\gamma +}^{2},\mu_{\gamma +},\mu_{\gamma -},\sigma_{\beta -}^{2},\sigma_{\gamma -}^{2}),\\ \begin{array}{lll} L & \propto & \left[Y_{n}|X_{n},\mu ,\beta ,\gamma ,\sigma_{\varepsilon }^{2}]\times [\mu ]\times [\mu_{\beta +}]\times [\sigma_{\beta +}^{2}]\times [\mu_{\beta -}]\times [\sigma_{\beta -}^{2}]\times [w_{\beta +},w_{\beta -}\right]\\ & & \times [\beta |w_{\beta +},w_{\beta -},\mu_{\beta +},\sigma_{\beta +}^{2},\mu_{\beta -},\sigma_{\beta -}^{2}]\times [\mu_{\gamma +}]\times [\sigma_{\gamma +}^{2}]\times [\mu_{\gamma -}]\times [\sigma_{\gamma -}^{2}]\\ & & \times [w_{\gamma +},w_{\gamma -}]\times [\gamma |w_{\gamma +},w_{\gamma -},\mu_{\gamma +},\sigma_{\gamma +}^{2},\mu_{\gamma -},\sigma_{\gamma -}^{2}]\times [\sigma_{\varepsilon }^{2}]\times [X_{n}]\\ & \propto & \sigma_{\varepsilon }^{-n-2}\exp\left\{-\sum_{i=1}^{n}\frac{{\left(Y_{i}-\mu -X_{i}\beta -Z_{i}\gamma \right)}^{2}}{2\sigma_{\varepsilon }^{2}}\right\}\times \exp\left(-\frac{\sigma_{\beta +}^{2}}{\varphi_{\beta +}}-\frac{\sigma_{\beta -}^{2}}{\varphi_{\beta -}}\right)\\ & & \times {\left(\sigma_{\beta +}^{2}\right)}^{-\theta_{\beta +}-1}\times {\left(\sigma_{\beta -}^{2}\right)}^{-\theta_{\beta -}-1}\times \exp\left(-\frac{\sigma_{\gamma +}^{2}}{\varphi_{\gamma +}}-\frac{\sigma_{\gamma -}^{2}}{\varphi_{\gamma -}}\right)\times {\left(\sigma_{\gamma +}^{2}\right)}^{-\theta_{\gamma +}-1}\\ & & \times {\left(\sigma_{\gamma -}^{2}\right)}^{-\theta_{\gamma -}-1}\times [\beta |w_{\beta +},w_{\beta -},\mu_{\beta +},\sigma_{\beta +}^{2},\mu_{\beta -},\sigma_{\beta -}^{2}]\times I\left[w_{\beta +}+w_{\beta -}\leq c\frac{\sqrt{n}}{p_{\beta }}\right]\\ & & \times [\gamma |w_{\gamma +},w_{\gamma -},\mu_{\gamma +},\sigma_{\gamma +}^{2},\mu_{\gamma -},\sigma_{\gamma -}^{2}]\times I\left[w_{\gamma +}+w_{\gamma -}\leq c\frac{\sqrt{n}}{p_{\gamma }}\right]\times [X_{n}]. \end{array} \end{array}$$

The distribution of ***X***_*n *_can be specified based on the available linkage map information [2]. The conditional distribution of $\left[\beta |w_{\beta +},w_{\beta -},\mu_{\beta +},\sigma_{\beta +}^{2},\mu_{\beta -},\sigma_{\beta -}^{2}\right]$ is a product of the prior distribution for each *β*_*j*_. Similarly, the conditional distribution of $\left[\gamma |w_{\gamma +},w_{\gamma -},\mu_{\gamma +},\sigma_{\gamma +}^{2},\mu_{\gamma -},\sigma_{\gamma -}^{2}\right]$ is a product of the prior distribution for each *γ*_*j*_. The priors of the hyperparameters, *θ*_*β*+_, *θ*_*γ*+_, *φ*_*β*+_, *φ*_*γ*+_, *θ*_*β*-_, *θ*_*γ*-_, *φ*_*β*- _and *φ*_*γ*-_, are specified to be as noninformative as possible.

#### Gibbs Sampling

Since the specified priors are conditionally conjugate, Bayesian variable selection can be implemented with a Gibbs sampling algorithm. We initialize the algorithm by imputing missing genotypic values based on the observed genotypes and linkage information. The initial value of *μ *is set as the mean of the observed trait values. Then, with individuals having fully observed trait values, each component of ***β ***and ***γ ***is initially estimated using recursive univariate regression. Other parameters, $w_{\beta +},w_{\beta -},\mu_{\beta +},\sigma_{\beta +}^{2},\mu_{\beta -}$ and $\sigma_{\beta -}^{2}$, are simply initialized based on the initial value of ***β***, and similarly, the initial values for $w_{\gamma +},w_{\gamma -},\mu_{\gamma +},\sigma_{\gamma +}^{2},\mu_{\gamma -}$, and $\sigma_{\gamma -}^{2}$ can be specified using the information from ***γ***. For example, we can initialize $\sigma_{\beta +}^{2}=\sigma_{\beta -}^{2}$ with an estimate from the initial value *β*, and then use max{#{*j *: *β*_*j *_> 2*σ*_*β*+_}, 1}/*p*_*β *_to initialize *w*_*β*+_.

Let ***X***_*i*,-*j *_be ***X***_*i *_excluding the *j*-th component, and define ***β***_-*j *_and ***γ***_-*j *_similarly. Based on the likelihood function in (4), the Gibbs sampler can be developed by recursively drawing the missing genotypic values, the missing trait values, and the model parameters from their full conditional posterior distributions as follows.

**Sample missing values: **Sample each missing genotypic value *X*_*ij *_from its full conditional posterior distribution,

$$[X_{ij}|Y_{i},X_{i,-j},\mu ,\beta ,\gamma ,\sigma_{\varepsilon }^{2}]\propto [Y_{i}|X_{i,-j},X_{ij},\mu ,\beta ,\gamma ,\sigma_{\varepsilon }^{2}]\times [X_{ij}|X_{i,j-1},X_{i,j+1}],$$

and then sample each missing trait value *Y*_*i *_from its full conditional posterior distribution [*Y*_*i*_*|X*_*i*_, *μ*, *β*, *γ*, $\sigma_{\varepsilon }^{2}$].

**Sample *μ*: **Sample *μ *from its full conditional posterior distribution,

$$\mu |Y_{n},X_{n},\beta ,\gamma ,\sigma_{\varepsilon }^{2}\sim N\left(\frac{1}{n}\sum_{i=1}^{n}(Y_{i}-X_{i}\beta -Z_{i}\gamma ),\frac{\sigma_{\varepsilon }^{2}}{n}\right).$$

**Sample *β *and *γ*: **Sample each *β*_*j *_and *γ*_*j *_from their full conditional posterior distributions,

[βj|Yn,Xn,μ,β−j,γ,wβ+,wβ−,σε2,σβ+2,σβ−2]~(1−w˜βj+−w˜βj−)δ(βj)+w˜βj+N+(μ˜βj+,σ˜βj+2)+w˜βj−N−(μ˜βj−,σ˜βj−2),[γj|Yn,Xn,μ,β,γ−j,wγ+,wγ−,σε2,σγ+2,σγ−2]~(1−w˜γj+−w˜γj−)δ(γj)+w˜γj+N+(μ˜γj+,σ˜γj+2)+w˜γj−N−(μ˜γj−,σ˜γj−2),

where ${\tilde{w}}_{\beta j+},{\tilde{w}}_{\beta j-},{\tilde{\mu }}_{\beta j+},{\tilde{\sigma }}_{\beta j+}^{2},{\tilde{\mu }}_{\beta j-}$, and ${\tilde{\sigma }}_{\beta j-}^{2}$ are specified in the APPENDIX. In addition, ${\tilde{w}}_{\gamma j+},{\tilde{w}}_{\gamma j-},{\tilde{\mu }}_{\gamma j+},{\tilde{\sigma }}_{\gamma j+}^{2},{\tilde{\mu }}_{\gamma j-}$, and ${\tilde{\sigma }}_{\gamma j-}^{2}$ can be obtained similarly.

**Sample ***w*_*β*+_, *w*_*γ*+_, *w*_*β*-_, **and ***w*_*γ*-_: These parameters can be sampled from the conditional posterior distributions,

$$\begin{array}{l} (w_{\beta +},w_{\beta -},1-w_{\beta +}-w_{\beta -})|\beta \\ \sim Dirichlet({\tilde{p}}_{\beta +}+1,{\tilde{p}}_{\beta -}+1,p_{\beta }-{\tilde{p}}_{\beta +}-{\tilde{p}}_{\beta -}+1),w_{\beta +}+w_{\beta -}\leq \frac{c\sqrt{n}}{p_{\beta }},\\ (w_{\gamma +},w_{\gamma -},1-w_{\gamma +}-w_{\gamma -})|\gamma \\ \sim Dirichlet({\tilde{p}}_{\gamma +}+1,{\tilde{p}}_{\gamma -}+1,p_{\gamma }-{\tilde{p}}_{\gamma +}-{\tilde{p}}_{\gamma -}+1),w_{\gamma +}+w_{\gamma -}\leq \frac{c\sqrt{n}}{p_{\gamma }}, \end{array}$$

where ${\tilde{p}}_{\beta +}$ = #{*β*_*j *_> 0 : 1 ≤ *j *≤ *p*_*β*_} and ${\tilde{p}}_{\beta -}$ = #{*β*_*j *_< 0 : 1 ≤ *j *≤ *p*_*β*_}; ${\tilde{p}}_{\gamma +}$ = #{*γ*_*j *_> 0 : 1 ≤ *j *≤ *p*_*γ*_} and ${\tilde{p}}_{\gamma -}$ = #{*γ*_*j *_< 0 : 1 ≤ *j *≤ *p*_*γ*_}.

**Sample **$\sigma_{\varepsilon }^{2},\sigma_{\beta +}^{2},\sigma_{\gamma +}^{2},\sigma_{\beta -}^{2}$**, and **$\sigma_{\gamma -}^{2}$**: **With conditionally conjugate priors, the posterior for all variance parameters are still inverse gamma distributions. Specifically,

$$\begin{array}{l} \sigma_{\varepsilon }^{2}|Y_{n},X_{n},\mu ,\beta ,\gamma \sim IG\left(\frac{n}{2},\frac{2}{\sum_{i=1}^{n}{\left(Y_{i}-\mu -X_{i}\beta -Z_{i}\gamma \right)}^{2}}\right),\\ \sigma_{\beta +}^{2}|\beta \sim IG\left(\theta_{\beta +}+\frac{{\tilde{p}}_{\beta +}}{2},\frac{2}{\frac{2}{\varphi_{\beta +}}+\sum_{j=1}^{p_{\beta }}\beta_{j}^{2}I[\beta_{j}>0]}\right),\\ \sigma_{\gamma +}^{2}|\gamma \sim IG\left(\theta_{\gamma +}+\frac{{\tilde{p}}_{\gamma +}}{2},\frac{2}{\frac{2}{\varphi_{\gamma +}}+\sum_{j=1}^{p_{\gamma }}\gamma_{j}^{2}I[\gamma_{j}>0]}\right),\\ \sigma_{\beta -}^{2}|\beta \sim IG\left(\theta_{\beta -}+\frac{{\tilde{p}}_{\beta -}}{2},\frac{2}{\frac{2}{\varphi_{\beta -}}+\sum_{j=1}^{p_{\beta }}\beta_{j}^{2}I[\beta_{j}<0]}\right),\\ \sigma_{\gamma -}^{2}|\gamma \sim IG\left(\theta_{\gamma -}+\frac{{\tilde{p}}_{\gamma -}}{2},\frac{2}{\frac{2}{\varphi_{\gamma -}}+\sum_{j=1}^{p_{\gamma }}\gamma_{j}^{2}I[\gamma_{j}<0]}\right). \end{array}$$

#### Bayesian Inference

For each variable in model (1), one pair of parameters is used to select the corresponding variable. They are, for the *j*-th additive effect, the posterior probabilities *w*_*β j*+ _= *P *(*β*_*j *_> 0|**Y**_*n*_, **X**_*n*_) and *w*_*β j*- _= *P *(*β*_*j *_< 0|**Y**_*n*_, **X**_*n*_). With the full conditional posterior distribution of *β*_*j *_and all the notations in the APPENDIX, we have

$$\begin{array}{cc} w_{\beta j+}=E[{\tilde{w}}_{\beta j+}|Y_{n},X_{n}], & w_{\beta j-}=E[{\tilde{w}}_{\beta j-}|Y_{n},X_{n}]. \end{array}$$

Therefore, the two parameters *w*_*β j*+ _and *w*_*β j*- _can be estimated with the Markov chains of ${\tilde{w}}_{\beta j+}$ and ${\tilde{w}}_{\beta j-}$ drawn from the above Gibbs sampler. If and only if both *w*_*β j*+ _and *w*_*β j*- _are less than 0.5, the median of the posterior distribution of *β*_*j *_is zero. Similarly, the posterior probabilities *w*_*γ j*+ _= *P *(*γ*_*j *_> 0|***Y***_*n*_, ***X***_*n*_) and *w*_*γ j*- _= *P *(*γ*_*j *_< 0|***Y***_*n*_, ***X***_*n*_) can be estimated with the Markov chains of ${\tilde{w}}_{\gamma j+}$ and ${\tilde{w}}_{\gamma j-}$ drawn from the above Gibbs sampler.

We propose to select variables twice under the above Bayesian framework for model (1). At the first step, we use a restrictive prior for each coefficient to ensure an identifiable Bayesian model and enforce to stochastically search for an optimal low-dimensional parameter subspace. We then rank the *j*-th additive effect based on max{*w*_*β j*+_, *w*_*β j*-_}, and rank the *j*-th epistatic effect based on max{*w*_*γ j*+_, *w*_*γ j*-_}. The top *c*$\sqrt{n}$ out of *p*_*β *_additive effects, and the top *c*$\sqrt{n}$ out of *p*_*γ *_epistatic effects are selected, respectively. At the second step, we select variables out of those selected *c*$\sqrt{n}$ additive effects and *c*$\sqrt{n}$ epistatic effects, under the above Bayesian framework for model (1). Obviously, we have a non-restrictive prior for each coefficient at the second step, and therefore avoid possible over-penalization due to restrictive priors.

Following Jeffreys [49,50], we test the hypothesis *H*_0 _: *β*_*j *_= 0 vs. *H*_1_: *β*_*j *_≠ 0 on the basis of the Bayes factor, which was defined as

$$B_{10}(\beta_{j})=\frac{P(Data|\beta_{j}\neq 0)}{P(Data|\beta_{j}=0)}=\frac{P(\beta_{j}\neq 0|Data)}{P(\beta_{j}=0|Data)}\times \frac{\pi (\beta_{j}=0)}{\pi (\beta_{j}\neq 0)}=\frac{w_{\beta j+}+w_{\beta j-}}{1-w_{\beta j+}-w_{\beta j-}},$$

where *π *(*β*_*j *_= 0) and *π *(*β*_*j *_≠ 0) are the *a priori *probabilities, and the last equality follows the fact that *π *(*β*_*j *_= 0) = *π *(*β*_*j *_≠ 0) at the second step of our Bayesian Classification. As suggested by Jeffreys [50], a *B*_10 _(*β*_*j*_) with value between 1 and $\sqrt{10}$ ≈ 3.2 provides "not worth more than a bare mention" evidence against *H*_0_; a *B*_10 _(*β*_*j*_) with value from $\sqrt{10}$ to 10 provides "substantial" evidence against *H*_0_; a *B*_10 _(*β*_*j*_) with value from 10 to 100 provides "strong" evidence against *H*_0_; and a *B*_10 _(*β*_*j*_) with value larger than 100 provides "decisive" evidence against *H*_0_. Similarly, we can test the hypothesis *H*_0_: *γ*_*j *_= 0 vs. *H*_1_: *j *≠ 0 using the following Bayes factor

(5) $$B_{10}(\gamma_{j})=\frac{w_{\gamma j+}+w_{\gamma j-}}{1-w_{\gamma j+}-w_{\gamma j-}}.$$

## Authors' contributions

MZ and DZ both conceived the study and developed the method. DZ wrote the MATLAB^® ^code and did the simulation study. MZ analyzed the real data. MTW participated in conceptual development, writing, reviewing and editing the manuscript. All authors read and approved the final manuscript.

## Appendix

**Fully Conditional Posterior Distribution of ***β*_*j*_

For each *j *= 1, ⋯, *p*_*β*_, the fully conditional posterior distribution of *β*_*j *_is

$$\begin{array}{l} \beta_{j}|Y_{n},X_{n},\mu ,\beta_{-j},\gamma ,w_{\beta +},w_{\beta -},\sigma_{\varepsilon }^{2},\sigma_{\beta +}^{2},\sigma_{\beta -}^{2}\\ \sim (1-{\tilde{w}}_{\beta j+}-{\tilde{w}}_{\beta j-})\delta_{\left(0\right)}+{\tilde{w}}_{\beta j+}N_{+}({\tilde{\mu }}_{\beta j+},{\tilde{\sigma }}_{\beta j+}^{2})+{\tilde{w}}_{\beta j-}N_{-}({\tilde{\mu }}_{\beta j-},{\tilde{\sigma }}_{\beta j-}^{2}), \end{array}$$

where the updated parameter values are

$$\begin{array}{lll} {\tilde{\mu }}_{\beta j+} & = & \sigma_{\beta +}^{2}\sum_{i=1}^{n}X_{ij}(Y_{i}-\mu -X_{i,-j}\beta_{-j}-Z_{i}\gamma )/\left(\sigma_{\varepsilon }^{2}+\sigma_{\beta +}^{2}\sum_{i=1}^{n}X_{ij}^{2}\right),\\ {\tilde{\sigma }}_{\beta j+}^{2} & = & \sigma_{\beta +}^{2}\sigma_{\varepsilon }^{2}/\left(\sigma_{\varepsilon }^{2}+\sigma_{\beta +}^{2}\sum_{i=1}^{n}X_{ij}^{2}\right),\\ {\tilde{\mu }}_{\beta j-} & = & \sigma_{\beta -}^{2}\sum_{i=1}^{n}X_{ij}(Y_{i}-\mu -X_{i,-j}\beta_{-j}-Z_{i}\gamma )/\left(\sigma_{\varepsilon }^{2}+\sigma_{\beta -}^{2}\sum_{i=1}^{n}X_{ij}^{2}\right),\\ {\tilde{\sigma }}_{\beta j-}^{2} & = & \sigma_{\beta -}^{2}\sigma_{\varepsilon }^{2}/\left(\sigma_{\varepsilon }^{2}+\sigma_{\beta -}^{2}\sum_{i=1}^{n}X_{ij}^{2}\right),\\ {\tilde{w}}_{\beta j+} & = & \frac{{\tilde{\sigma }}_{\beta j+}}{\sigma_{\beta +}}\Phi \left(\frac{{\tilde{\mu }}_{\beta j+}}{{\tilde{\sigma }}_{\beta j+}}\right)/[\frac{1-w_{\beta +}-w_{\beta -}}{2w_{\beta +}}\exp\left(-\frac{{\tilde{\mu }}_{\beta j+}^{2}}{2{\tilde{\sigma }}_{\beta j+}^{2}}\right)+\frac{{\tilde{\sigma }}_{\beta j+}}{\sigma_{\beta +}}\\ & & \times \Phi \left(\frac{{\tilde{\mu }}_{\beta j+}}{{\tilde{\sigma }}_{\beta j+}}\right)+\frac{w_{\beta -}{\tilde{\sigma }}_{\beta j-}}{w_{\beta +}\sigma_{\beta -}}\Phi \left(-\frac{{\tilde{\mu }}_{\beta j-}}{{\tilde{\sigma }}_{\beta j-}}\right)\exp\left(\frac{{\tilde{\mu }}_{\beta j-}^{2}}{2{\tilde{\sigma }}_{\beta j-}^{2}}-\frac{{\tilde{\mu }}_{\beta j+}^{2}}{2{\tilde{\sigma }}_{\beta j+}^{2}}\right)],\\ {\tilde{w}}_{\beta j-} & = & \frac{{\tilde{\sigma }}_{\beta j-}}{\sigma_{\beta -}}\Phi \left(-\frac{{\tilde{\mu }}_{\beta j-}}{{\tilde{\sigma }}_{\beta j-}}\right)/[\frac{1-w_{\beta +}-w_{\beta -}}{2w_{\beta -}}\exp\left(-\frac{{\tilde{\mu }}_{\beta j-}^{2}}{2{\tilde{\sigma }}_{\beta j-}^{2}}\right)+\frac{{\tilde{\sigma }}_{\beta j-}}{\sigma_{\beta j-}}\\ & & \times \Phi \left(-\frac{{\tilde{\mu }}_{\beta j-}}{{\tilde{\sigma }}_{\beta j-}}\right)+\frac{w_{\beta +}{\tilde{\sigma }}_{\beta j+}}{w_{\beta -}\sigma_{\beta +}}\Phi \left(\frac{{\tilde{\mu }}_{\beta j+}}{{\tilde{\sigma }}_{\beta j+}}\right)\exp\left(\frac{{\tilde{\mu }}_{j+}^{2}}{2{\tilde{\sigma }}_{\beta j+}^{2}}-\frac{{\tilde{\mu }}_{\beta j-}^{2}}{2{\tilde{\sigma }}_{\beta j-}^{2}}\right)]. \end{array}$$
